# USP29 enhances chemotherapy-induced stemness in non-small cell lung cancer via stabilizing Snail1 in response to oxidative stress

**DOI:** 10.1038/s41419-020-03008-5

**Published:** 2020-09-23

**Authors:** Yueguang Wu, Yingqiu Zhang, Duchuang Wang, Yang Zhang, Jinrui Zhang, Yayun Zhang, Lu Xu, Taishu Wang, Shanshan Wang, Qingqing Zhang, Fang Liu, Mohamed Y. Zaky, Qiong Li, Qianhui Sun, Dong Guo, Shuyan Liu, Lijuan Zou, Qingkai Yang, Han Liu

**Affiliations:** 1 The Second Affiliated Hospital, Institute of Cancer Stem Cell, Dalian Medical University, Dalian, China; 2 Department of Pathology, Dalian Medical University, Dalian, China; 3 Molecular Physiology Division, Department of Zoology, Faculty of Science, Beni-Suef University, Beni-Suef, Egypt; 4 Department of Radiation Oncology, Second Affiliated Hospital, Dalian Medical University, Dalian, China

**Keywords:** Deubiquitylating enzymes, Non-small-cell lung cancer

## Abstract

Chemotherapy remains an essential part of diverse treatment regimens against human malignancies. However, recent progressions have revealed a paradoxical role of chemotherapies to induce the cancer stem cell-like features that facilitate chemoresistance and tumor dissemination, with the underlying mechanisms underinvestigated. The zinc-finger transcription factor Snail1 is a central regulator during the epithelial-mesenchymal transition process and is closely implicated in cancer progression. Snail1 expression is strictly regulated at multiple layers, with its stability governed by post-translational ubiquitylation that is counterbalanced by the activities of diverse E3 ligases and deubiquitylases. Here we identify the deubiquitylase USP29 as a novel stabilizer of Snail1, which potently restricts its ubiquitylation in a catalytic activity-dependent manner. Bioinformatic analysis reveals a reverse correlation between USP29 expression and prognosis in lung adenocarcinoma patients. USP29 is unique among Snail1 deubiquitylases through exhibiting chemotherapy-induced upregulation. Mechanistically, oxidative stresses incurred by chemotherapy stimulate transcriptional activation of USP29. USP29 upregulation enhances the cancer stem cell-like characteristics in lung adenocarcinoma cells to promote tumorigenesis in athymic nude mice. Our findings uncover a novel mechanism by which chemotherapy induces cancer stemness and suggest USP29 as a potential therapeutic target to impede the development of chemoresistance and metastasis in lung adenocarcinoma.

## Introduction

Cytotoxic chemotherapy represents one classic mode of cancer treatment, which effectively prolonged overall survivals of cancer patients over the decades. Although its adverse side effects are mainly considered as cytotoxicity-related, accumulating evidences have started to associate chemotherapeutic treatments with enhanced cancer stemness that leads to drug resistance and tumor dissemination^1^. Convincing evidences have been demonstrated that both host and tumor cells are capable of eliciting responses to promote tumor survival, cultivate chemoresistance, and assist metastasis^1–5^.

The zinc-finger transcription factor SNAI1, more commonly known as Snail1, functions as a master regulator to promote the epithelial-mesenchymal transition (EMT) process through both repression of E-cadherin expression and the activated transcriptions of a panel of mesenchymal genes associated with invasive properties^6,7^. In addition to critical roles in embryonic development and tissue regeneration, EMT has been revealed to be intimately involved in the stemness and invasiveness of malignancies^8–10^. Accordingly, Snail1 expression was closely implicated in the progression of multiple types of cancer, generally correlating with elevated cancer stem cell properties, increased chemoresistance, metastasis, and poor clinical outcomes^11–13^.

As a pivotal factor governing the expressions of numerous downstream genes, Snail1 levels are also dynamically regulated both transcriptionally and post-translationally. Regarding the post-translational modification of Snail1 by ubiquitylation, multiple E3 ligases including β-TRCP, FBXL14, FBXO11, FBXL5, and FBXO45, were reported to be capable of adding ubiquitin moieties covalently onto Snail1 to cause proteasomal degradation, rendering it a labile transcription factor^14–18^. As countervailing measures to maintain the homeodynamics of Snail1 levels, recent investigations have revealed the involvements of several deubiquitylase (DUB) activities to stabilize Snail1 by removing attached ubiquitin molecules. DUB3 (official symbol USP17L2) was shown to promote breast cancer invasion and metastasis via stabilizing Snail1 in a CDK4/6 activity-dependent manner^19,20^. OTUB1 was observed to modulate Snail1 levels to enhance the metastasis of esophageal squamous carcinoma^21^. Furthermore, USP27X was reported to stabilize Snail1 levels and hence required for TGFβ-induced EMT and fibroblast activation^22^.

DUBs encompass a family of about 90 cysteine proteases and metalloproteases that is generally divided into seven subfamilies, including USP (ubiquitin-specific protease), UCH (ubiquitin C-terminal hydrolase), OTU (ovarian tumor protease), JAMM (Jab1/Pad1/MPN-domain-containing metallo-enzyme), Ataxin-3/Josephin (Ataxin-3/Josephin-domain-containing protein), MINDY (motif interacting with Ub-containing novel DUB family), and ZUP1^23–25^. Previous studies have revealed the distinct subcellular localizations of different DUBs, indicating their diverse functionalities^26,27^. Given that both ubiquitylation and deubiquitylation of Snail1 can take place in the nucleus, we screened a panel of nuclear DUBs to investigate their capabilities to regulate Snail1 levels^20,28,29^. Here, we report USP29 as a novel DUB that potently stabilized the protein levels of Snail1 through exerting DUB activity. Elevated USP29 expression associated with enhanced cancer stem cell properties in lung adenoma cells and poor prognosis in lung cancer. Importantly, USP29 levels were induced by chemotherapy and oxidative stress treatments, indicating a novel mechanism exploited by cancer cells to acquire drug resistance and enable metastasis in response to chemotherapies.

## Materials and methods

### Cell culture

Lung adenocarcinoma cell lines H1299, H1975, A549, and HCC827 were obtained from the American Type Culture Collection (ATCC) and cultured in RPMI-1640 media (Gibco). HEK293T, U2OS, and HeLa cells were generous gifts from Prof. Haixin Lei (Dalian Medical University) and maintained in Dulbecco’s modified Eagle’s medium (DMEM, Gibco). Fetal bovine serum (10%, Gibco) and antibiotics (1% penicillin/streptomycin, Thermo Fisher) were routinely added into RPMI-1640 and DMEM base media to prepare full growth media. Cells were placed at a humidified atmosphere in an incubator (Thermo, 3111) maintaining a constant CO_2_ concentration of 5% at 37 °C. Plastic wares for cell culture were purchased from Guangzhou Jet Bio-Filtration Co., Ltd. All cell lines were routinely tested for mycoplasma contamination.

### Plasmids and DNA transfection

The DUB expression constructs (Flag-HA tagged) have been described previously by Sowa et al.^30^, which were obtained from Addgene and selected DUBs were subcloned into pEGFP-C1 vectors. Wild-type Snail1 and its 6SA mutant constructs were reported previously by Zhou et al.^18^ and were obtained from Addgene. USP29 truncation constructs were generated with standard PCR into pEGFP-C1 backbone, while USP29 point mutations were introduced with site-directed mutagenesis (QuikChange, Agilent). Plasmid transfection was carried out using Lipofectamine 3000 reagent (ThermoFisher) as per manufacturer’s instructions.

### Antibodies and other reagents

Rabbit anti-USP29 (HPA021064), mouse anti-Flag M2 (F1804), and mouse anti-α-Tubulin antibodies were purchased from Sigma; mouse anti-Snail1 (3895S) antibody was obtained from Cell Signaling Technology; mouse anti-GFP (11814460001) antibody was obtained from Roche; mouse anti-HA tag (MMS-101P) antibody was obtained from Covance. Goat anti-mouse and anti-rabbit secondary antibodies (680 or 800 nm infrared-labeled) were obtained from LICOR. Alexa Fluor® 488 or 594 labeled immunofluorescence secondary antibodies were purchased from Invitrogen. Hoechst 33342 was purchased from Life Technologies. The chemotherapeutic agents doxorubicin and paclitaxel as well as verapamil hydrochloride were obtained from Dalian Meilun Biotechnology Co., Ltd. Cycloheximide was purchased from MP Biomedicals. Bortezomib (PS-341) was purchased from Selleckchem (Houston, TX, USA).

### Western blotting

To prepare protein lysates, treated cells were PBS washed and lysed using the “RIPA” buffer as previously described^31^. Protein samples were spun at 13, 000 rpm in a chilled benchtop centrifuge (Eppendorf) to remove particulates. Protein concentrations were measured using the Bradford assay. In SDS-PAGE analysis, same quantities of protein samples per condition were mixed with sample buffer and boiled, before gel separation using the Bio-Rad system. Samples were then wet transferred to nitrocellulose membranes (Merck Millipore, 0.45 μm). Following PBS washes, membranes were firstly blocked in 5% skimmed milk for 2 h at room temperature, followed by primary antibody incubation overnight in the cold room. The membranes were then PBS washed and incubated with secondary antibodies for 2 h at room temperature in the dark. After PBS washes, the membranes were finally detected using an Odyssey infrared imager (LICOR).

### Immunoprecipitation and co-immunoprecipitation

HEK293T cells were transfected with plasmids expressing GFP-USP29 and Flag-Snail1 as indicated, before harvest for protein lysate preparation using the Triton X-100 lysis buffer (50 mM Tris-HCl pH 7.4, 150 mM NaCl, 1 mM EDTA, and 1% Triton X-100). Experimental procedures were followed as described previously^32^. In brief, after protein concentration determination using the Bradford assay, one milligram of lysate per condition was incubated with protein G-agarose (Roche) and target antibody for 4 h at 4 °C. Then the agarose beads were washed three times using wash buffer (50 mM Tris-HCl pH 7.4, 150 mM NaCl, and 0.1% Triton X-100), prior to protein elution using 1.5× SDS-PAGE sample buffer. Immunoprecipitation samples were finally analyzed by routine Western blotting.

### Immunofluorescence assay

Cultured human osteosarcoma U2OS cells were seeded onto glass coverslips in 6-well plates and placed in the incubator for overnight. The following day cells were transfected with plasmids expressing flag-tagged Snail1 (pCMV-Flag) together with various GFP-tagged USP29 constructs (pEGFP-C1 vectors, GFP alone as control) as indicated, using Lipofectamine™ 3000 transfection reagent (Invitrogen) as per manufacturer’s instructions. After 24 h of incubation, cells were washed with PBS and fixed in 4% (w/v) paraformaldehyde for 15 min. Then cells were permeabilized with 0.2% Triton X100 for 5 min, before incubation in 2% BSA for 30 min. Following primary antibody treatment for 1 h, the coverslips were washed using PBS and then incubated with secondary fluorescence antibody for 30 min in the dark. The cells were stained with DAPI, before coverslips mounted onto glass slides with Mowiol (Sigma). The slides were air-dried for overnight before examined by fluorescence microscopy (Olympus BX63, Japan).

### Generation of stable cell lines

To generate H1299 and H1975 cells stably expressing USP29, *USP29* was subcloned into pCDH vector that was then used together with psPAX2 and pMD2.G plasmids to co-transfect HEK293T cells using Lipofectamine™ 3000 transfection reagent (Invitrogen) for lentivirus preparation. After 48 h of treatment, lentiviruses (pCDH-USP29 and pCDH vector control) were collected and added separately into H1299 and H1975 cells cultured in 3.5 cm dishes. After 12 h, H1299 and H1975 cells were subjected to treatment with 2 μg/ml of puromycin to screen for positive expression cells. USP29 overexpression was confirmed by Western blotting and stable cell lines were routinely maintained in culture media supplemented with 2 μg/ml of puromycin throughout all experiments to keep positive expression.

### Flow cytometry

Cultured H1975-pCDH, H1975-pCDH-USP29, H1299-pCDH, and H1299-pCDH-USP29 were harvested and suspended in antibiotic-free RPMI-1640 media at a density of 10^6^ cells/ml in the medium. Two samples (2 ml each) were prepared from each cell line, with one set incubated with 200 μM of verapamil hydrochloride at 37 °C for 15 min to block drug efflux and the other one treated with the solvent. Then samples were incubated with 5 µg/ml of Hoechst 33342 for 90 min at 37 °C in the dark, during which period cells were resuspended every 10 min. Following 10 min incubation on ice, cells were spun down in a chilled centrifuge and resuspended in 0.5 ml of cold medium without antibiotics, before treatment with propidium iodide (2 μg/ml) on ice for 10 min. The samples were finally processed by flow cytometry using FACS Aria ll (BD Biosciences). All acquired data were analyzed using FlowJo software (version 7.6).

### Spheroid formation

Cultured H1975-pCDH, H1975-pCDH-USP29, H1299-pCDH, and H1299-pCDH-USP29 cells were seeded into 96-well plates (ultra-low attachment) at a density of 500 cells/well in the serum-free DMEM-F12 medium supplemented with basic fibroblast growth factor (20 ng/ml), epidermal growth factor (20 ng/ml), and B27 (2% v/v). Cells were maintained in the incubator to allow spheroid formation, with images captured under a phase-contrast microscope (Leica, Germany) at day 8 and 15. The sizes of spheroids were quantified using the ImageJ software.

### Transwell assay

H1299 and H1975 cells stably transfected with control and USP29-expressing vectors were detached from the culture dish by trypsinization. Cells were washed and resuspended in serum-free culture medium, before 30,000 cells from each condition were seeded separately into the upper chambers of the Transwell plate (Corning), while the lower chambers were filled with 600 μl of full growth medium. Following a 10 h incubation in the cell incubator, migrated cells were fixed with methanol prior to staining using 1% crystal violet for 15 min. The plate was dried and examined under an inverted microscope (Leica, DMI4000B). Captured images were analyzed with the ImageJ software.

### RNA extraction and RT-PCR

H1299 and H1975 stable cell lines were cultured in 3.5 cm dishes and each plate was harvested using 0.5 ml of TRIzol reagent (Invitrogen) as per manufacturer’s instructions. The quality of RNA preparations was confirmed by agarose gel electrophoresis, and the concentrations were determined using the Nanodrop equipment (Thermo). Five hundred nanograms of total RNA from each condition were used as templates for reverse transcription using the PrimeScript Reverse Transcription kit (TaKaRa), and then generated cDNA was used for semi-quantitative PCR assays using target-specific primer pairs that were listed in Supplementary Table 1.

### Xenograft mouse model

Experimental procedures carried out for animal studies were approved by the Institutional Animal Care and Use Committee at Dalian Medical University. Female nude mice (BALB/c background, 4–6 weeks) were obtained from Vital River Laboratories (Beijing, China) and housed under sterile conditions throughout experiments. Cultured H1299-pCDH (control) and H1299-pCDH-USP29 cells were harvested and resuspended in PBS solution to reach 1 million cells per 0.1 ml of PBS. Nude mice were randomized into two groups (5 mice per group), which were not blinded to investigators and subjected to subcutaneous inoculation of H1299-pCDH or H1299-pCDH-USP29 cells separately (900, 000 cells per mouse). The sizes of H1299-pCDH and H1299-pCDH-USP29 xenografts were measured every other day with vernier caliper. Tumor volume was calculated using the formula: (long axis) × (short axis)^2^ × 0.5. Mice were sacrificed 30 days after inoculation, and resected tumor xenografts were processed for Western blotting analysis.

### Statistical analysis

Experiments were routinely performed with three biological repeats unless specified. To determine the statistical difference between test groups, two-tailed Student’s *t*-tests were conducted to calculate *p* values using the GraphPad Prism software (version 7), with *p* values < 0.05 considered as statistically significant. Experimental data were illustrated as mean ± standard error of the mean (SEM).

## Results

### Nuclear DUB screening identifies USP29 as a potent Snail1 regulator

**Fig. 1:**
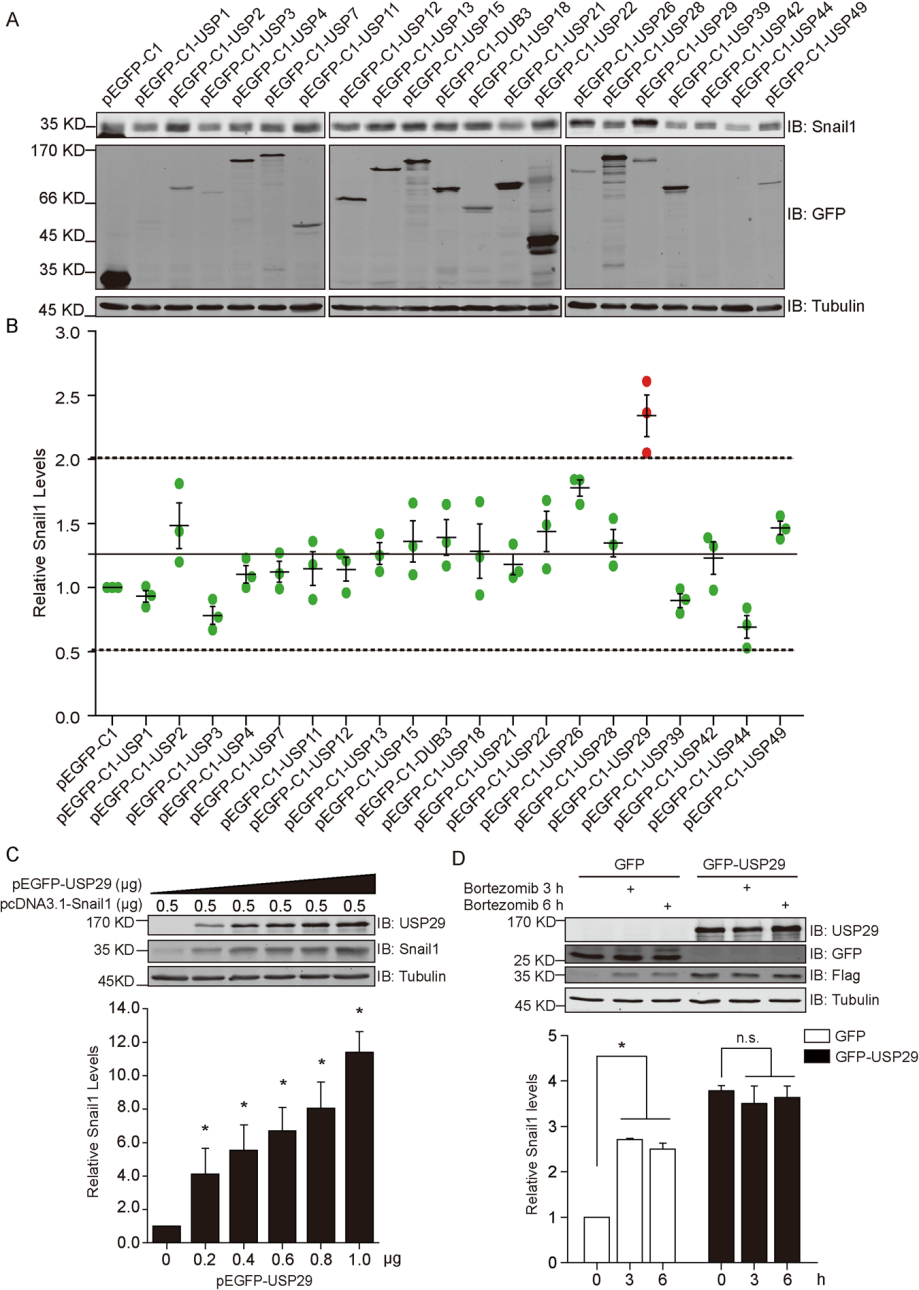
USP29 is a novel Snail1 regulator that potently stabilizes its levels. **a** HEK293T cells were co-transfected with constructs expressing Flag-Snail1 and various GFP-DUBs as indicated. After 24 h, the expression levels of Snail1 and DUBs were assessed by Western blotting. Tubulin was probed to confirm equal loading. Images are representative from three independent experiments. **b** Densitometry analysis of Snail1 band intensities from A summarizing three biological repeats, with pEGFP-C1 control set as 1. The data points were analyzed by Gaussian distribution showing mean (solid line) and 95% Confidence Interval (dashed lines). **c** HEK293T cells were co-transfected with Flag-Snail1 expressing plasmid at constant amounts and pEGFP-USP29 construct with escalating doses ranging from 0 to 1 μg, prior to Western blotting analysis with indicated antibodies. Below column chart shows the relative quantification of Snail1 expression (*n* = 3). **d** HEK293T cells co-transfected with Flag-Snail1 and pEGFP-C1 control or pEGFP-USP29 constructs were treated with the proteasomal inhibitor bortezomib (200 nM) for 3 and 6 h before cell lysis and Western blotting analysis using indicated antibodies. Below column chart shows the relative quantification of Snail1 levels. All error bars represent the standard error of the mean (SEM), with *p-value < 0.05 and n.s. representing not significant.

The subcellular localizations of DUBs likely indicate their functional implications. Taking advantage of the accumulating evidence on intracellular DUB locations, we collated a panel of nuclear-localized DUBs, including 13 exclusively nucleus-localized and 4 residing in both cytoplasm and nucleus (USP4, USP13, USP15, and USP21), supplemented with three cytoplasmic DUBs (USP2, USP12, and USP18) as controls^25,26^. These DUBs were tagged with GFP at N-termini and co-expressed with flag-tagged Snail1 in HEK293T cells. Quantification results from Western densitometry revealed that the majority of DUBs tested (16 out of 20) elevated Snail1 levels while compared to GFP control (Fig. 1a, b). Therefore, these observations call for caution in the interpretation of data with exogenous overexpression of DUB and potential substrate. Nevertheless, when these data points were considered as a Gaussian distribution, the statistic test apparently identified an outlier that significantly differed from the others, which represented USP29 (Fig. 1b). Relative to GFP control, the overexpression of GFP-tagged USP29 caused an on average over twofolds increase in Snail1 levels (Fig. 1b).

Subsequent validation of the initial screening revealed a dose-dependent effect of USP29 expression towards Snail1 stabilization (Fig. 1c). Since it is well recognized that the degradation of Snail1 is mediated by the proteasomes, we blocked proteasome activity using its specific inhibitor bortezomib (PS-341) to examine the influence on Snail1 expression. As illustrated in Fig. 1d, with 3 and 6 h of bortezomib treatment, we readily observed elevated Snail1 levels in GFP-expressing control cells but recorded no significant change of Snail1 amounts in those containing GFP-USP29, indicative of limited proteolysis and thus restricted ubiquitylation of Snail1 in the presence of this DUB.

### USP29 interacts with Snail1 and requires catalytic activity to stabilize Snail1

**Fig. 2:**
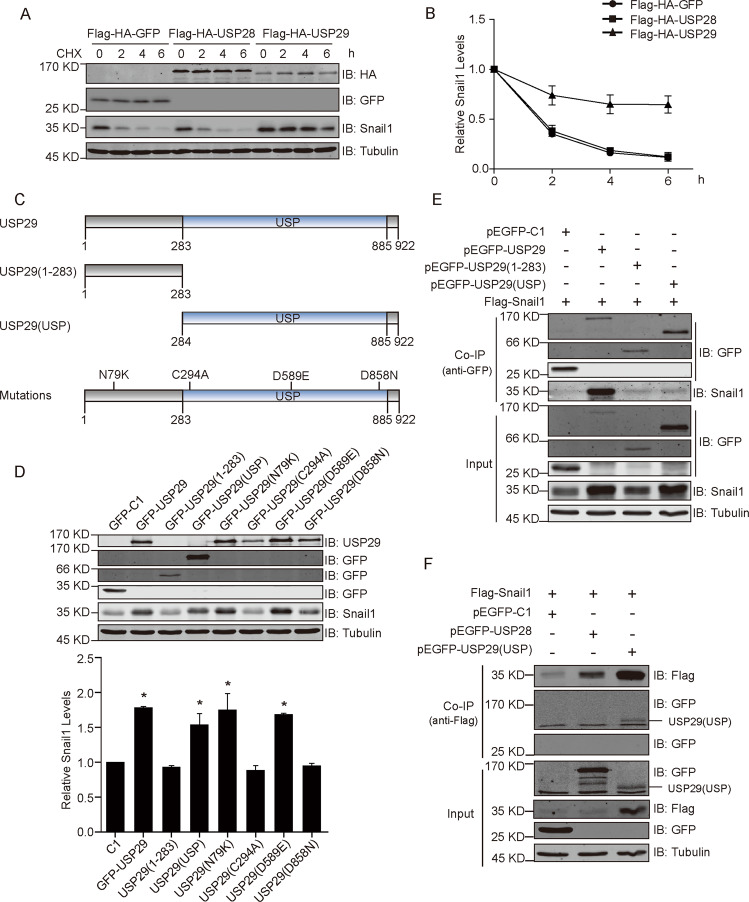
USP29 interacts with Snail1 and requires catalytic USP domain to stabilize Snail1. **a** HEK293T cells were co-transfected with Flag-Snail1 vector along with Flag-HA-GFP, Flag-HA-USP28, or Flag-HA-USP29 constructs for 24 h. Cells were treated with cycloheximide (100 μg/ml) to stop protein synthesis for indicated times and lysed for Western blotting assays with indicated antibodies. **b** turnover curves show the relative amounts of Snail1 at each time point from (**a**) (*n* = 3). **c** schematic illustration of the domain structure in USP29 and corresponding truncations with indicated amino acid sequences. **d** Flag-Snail1 and various GFP-USP29 constructs were co-transfected into HEK293T cells for 24 h, and then cell lysates were analyzed with Western blotting using indicated antibodies. Below column chart shows the relative quantification of Snail1 levels (*n* = 3). **e** HEK293T cells were co-transfected with indicated combinations of Flag-Snail1 and diverse USP29 constructs for 24 h. GFP or GFP-tagged USP29 variants were immunoprecipitated with anti-GFP antibodies. Samples were processed for Western blotting assays to probe for co-immunoprecipitated Snail1. **f** Flag-tagged Snail1 was immunoprecipitated from HEK293T cell lysates following co-transfections of Flag-Snail1 with pEGFP, pEGFP-USP28, or pEGFP-USP29-USP constructs. Immunoprecipitates were analyzed by Western blotting using indicated antibodies. All error bars represent the standard error of the mean (SEM), with *p value < 0.05 and n.s. representing not significant.

Consistently, in the subsequent cycloheximide chase experiment, the turnover of Snail1 was remarkably decelerated in GFP-USP29-expressing cells as compared to that in control cells containing GFP or GFP-USP28 (Fig. 2a, b). We then moved to investigate the potential interaction between USP29 and Snail1. According to the architecture of USP29, we generated two truncated versions including the N-terminus (amino acids 1–283) and the catalytic USP domain (amino acids 284-922 that extend to the C-terminus) (Fig. 2c). We also constructed four USP29 mutants with single amino acid substitutions, comprising the catalytic inactive C294A mutation and 3 mutations detected from sequencing analysis of cancer tissues (N79K, D589E, and D858N, retrieved from http://cbioportal.org)^33,34^ (Fig. 2c). Results from co-expression assays showed that the catalytic domain, N79K, D589E constructs effectively enhanced Snail1 levels, on a par with wild-type USP29, but the N-terminus, C294A, and D858N caused no significant change relative to GFP control (Fig. 2d). These observations imply the necessity of USP29 DUB activity in the regulation of Snail1, but the exact roles of USP29 mutations detected in cancer samples require further clarification.

To evaluate the interaction between Snail1 and USP29, we carried out a series of co-immunoprecipitation experiments. As shown in Fig. 2e, Snail1 was easily detected from USP29 immunoprecipitation but absent from the GFP control sample. Interestingly, Snail1 bands were also discernible from the immunoprecipitations of the N-terminus and catalytic domain variants, although much weaker compared to the wild-type lane, indicating that both truncated versions reserved certain degree of Snail1 binding but full-length protein exhibited efficient interaction with Snail1. Consistently, in the reverse assay wherein flag-Snail1 was immunoprecipitated, GFP-USP29(USP) was observed to be successfully co-precipitated, but not GFP or GFP-USP28 (Fig. 2f).

**Fig. 3:**
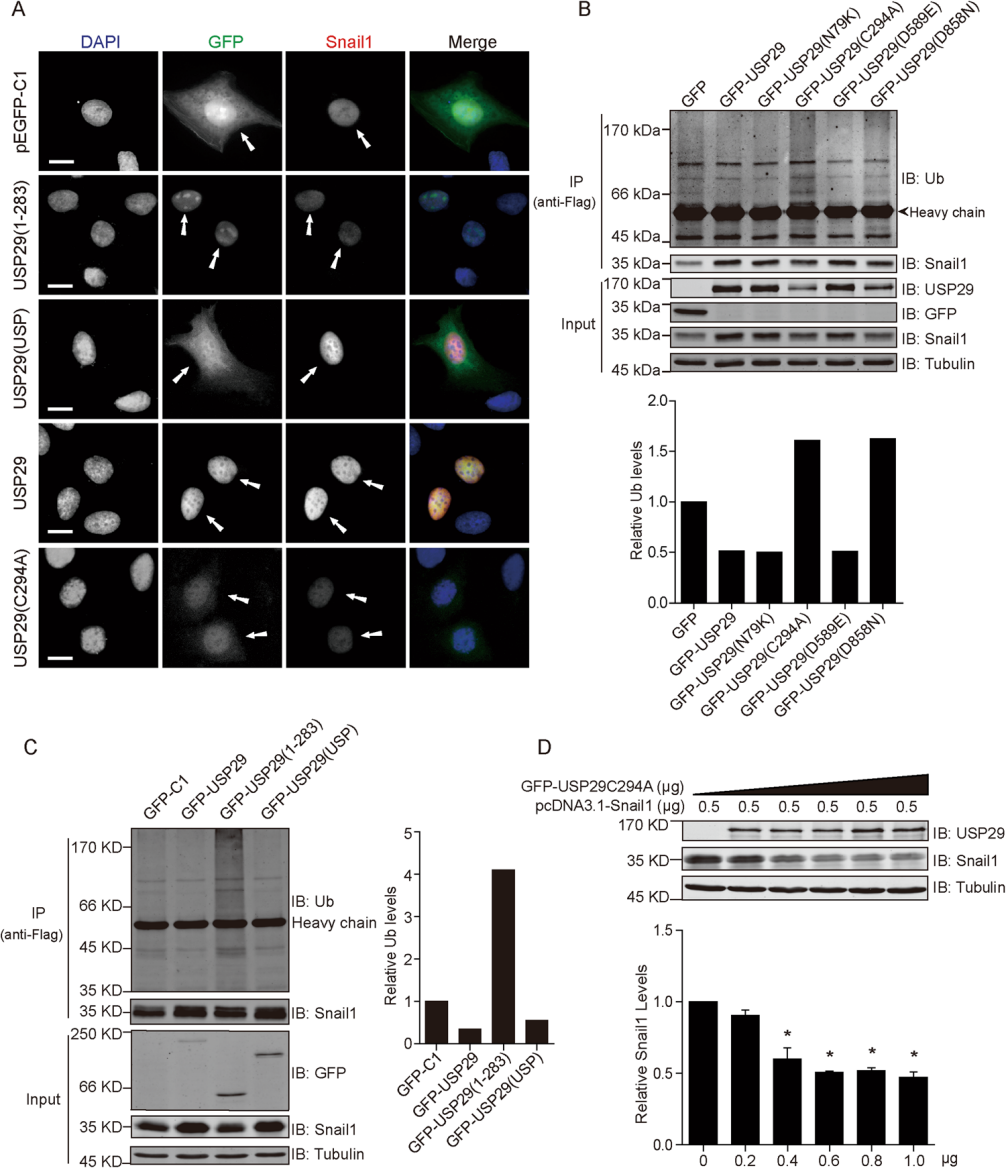
The Snail1-stabilizing effect of USP29 depends on its catalytic USP domain-mediated deubiquitylation. **a** U2OS cells were co-transfected with Flag-Snail1 and various GFP-USP29 constructs before immunofluorescence analysis. Flag-Snail1 was stained using anti-Flag antibody. GFP-USP29 variants were visualized through GFP. Micrographs show representative images from each condition captured under the same intensity settings, with DAPI staining showing nuclei. Scale bar = 10 µm. **b**, **c** HEK293T cells were co-transfected with Flag-Snail1 and indicated control or GFP-USP29 plasmids. Following bortezomib treatment (500 nM, 6 h) to block proteasomal degradation, Flag-Snail1 was immunoprecipitated from cell lysates using anti-Flag antibody. Immunoprecipitates were analyzed by Western blotting to examine the ubiquitylation status of Flag-Snail1 in different groups. Column charts show the relative quantification of the ubiquitin signal. **d** HEK293T cells were co-transfected with Flag-Snail1 (0.5 μg) and GFP-USP29-C294A (0–1 μg) constructs for 24 h. Cell lysates were analyzed by Western blotting with indicated antibodies. Below column chart shows the quantification data of Snail1 expression (*n* = 3). All error bars represent the standard error of the mean (SEM), with *p value < 0.05.

To further confirm the Snail1-stabilizing effect of various USP29 constructs, we conducted immunofluorescence assays by expressing GFP-tagged USP29 variants along with flag-Snail1 in U2OS cells. As demonstrated in Fig. 3a, full-length USP29 showed nuclear localization and correlated with elevated Snail1 staining; although the N-terminus version retained in the nucleus but failed to stabilize Snail; while the catalytic domain effectively enhanced nuclear Snail1 signal but showed a diffuse staining throughout the whole cell; and the catalytic inactive mutant (C294A) maintained nuclear localization without rescuing Snail1 levels. We then inspected the ubiquitylation status of Snail1 in the presence of different USP29 variants, and results from immunoprecipitation assays revealed that the DUB activity of USP29 reversely correlates with the ubiquitylation of Snail1 (Fig. 3b, c). In addition, the dose-escalating expression of the C294A USP29 mutant effectively suppressed Snail1 expression levels, suggesting a dominant-negative role of the catalytic inactive version that overwhelms the endogenous wild-type USP29 (Fig. 3d). Taken together, these results reaffirmed that the Snail1-stabilizing effect of USP29 was mediated through its DUB activity.

### USP29 correlates with enhanced stemness in lung adenocarcinoma cells

**Fig. 4:**
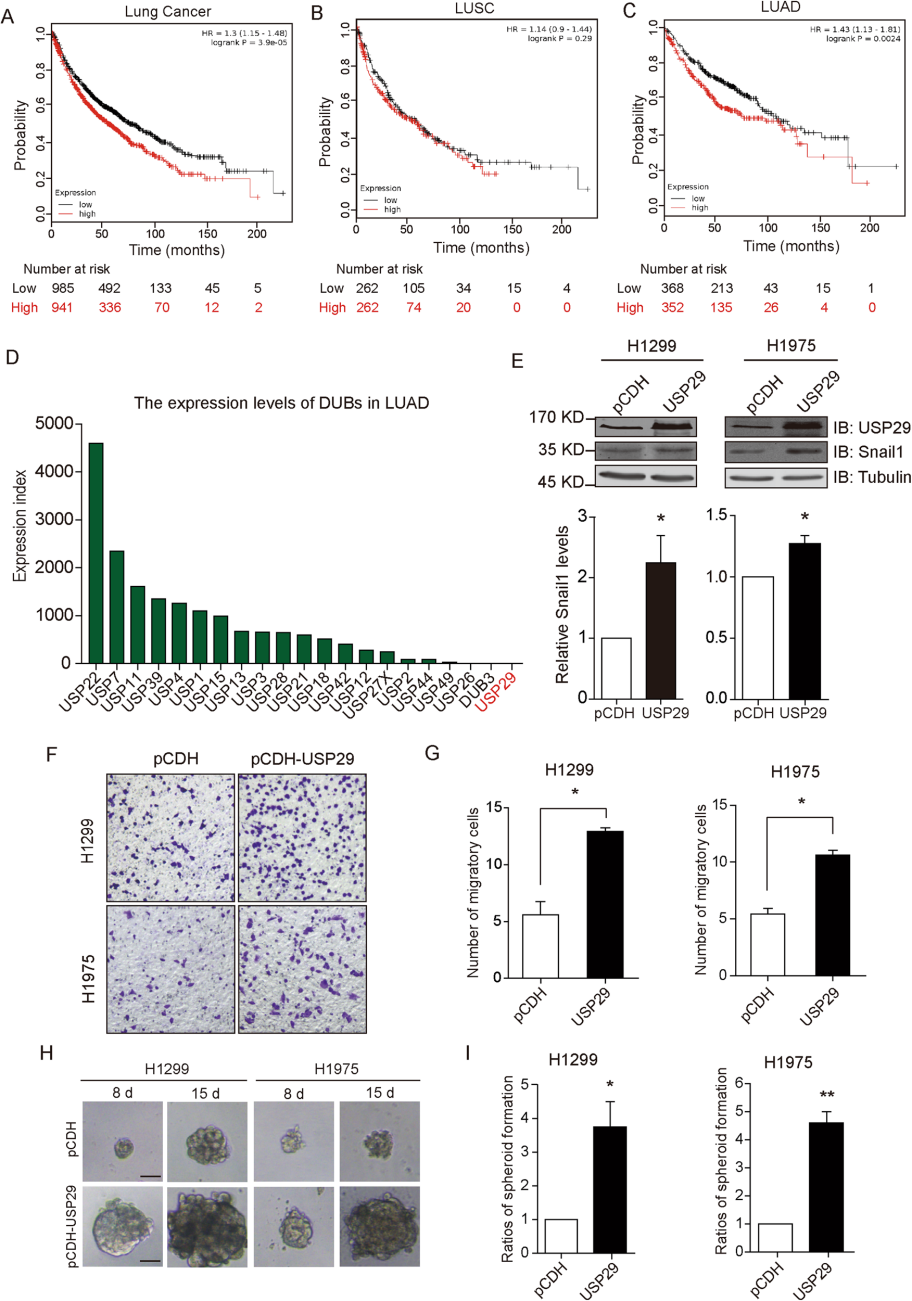
USP29 expression correlates with enhanced cancer stem cell features and poor prognosis in lung cancer. The prognosis data of lung cancer patients were retrieved from the KM-plotter website (www.kmplot.com), showing correlations between USP29 expression and overall survival in total lung cancer (**a**), lung squamous carcinoma (LUSC, **b**), and lung adenocarcinoma (LUAD, **c**). Patients were split by medians of USP29 expression. **d** RNA-seq data of indicated DUBs in 517 LUAD patients from TCGA (The Cancer Genome Atlas) were retrieved from the cBio Cancer Genomics Portal (http://cbioportal.org). Mean RPKM (reads per kilobase of exon per million reads mapped) of individual DUB genes was calculated to enable the quantitative comparison. **e** Characterization of H1299 and H1975 cells stably expressing USP29 in parallel with vector control stable cell lines. **f** Representative images from transwell assays using H1299 and H1975 cell lines stably transfected with pCDH and pCDH-USP29 as indicated. **g** Column charts show quantification data of migrated cells per microscopic view on average from three biologically independent experiments. (**h**), representative images of spheroids formed by H1299 and H1975 stable cell lines after day 8 and 15. Scale bar = 50 μm. **i** Column charts show relative quantification of spheroid numbers compared to pCDH control groups for H1299 and H1975 cells stably expressing USP29, respectively (*n* = 3). All error bars represent the standard error of the mean (SEM), with *p value < 0.05 and **p value < 0.01.

Having provided several lines of biochemical evidence that USP29 functioned to stabilize Snail1 levels through its deubiquitylation activity, we sought to search for clinical implications of USP29 expression in cancer tissues. Through interrogating bioinformatic databases, we observed a positive correlation of USP29 expression with poor prognoses in lung cancer patients as retrieved from the Kaplan–Meier Plotter website (Fig. 4a)^35^. Further analyses identifying different histological subtypes of lung cancer by discriminating between lung adenocarcinoma (*n* = 720) and squamous cell carcinoma (*n* = 524) revealed USP29 expression as a significant prognosis indicator in adenocarcinoma rather than squamous cell carcinoma (Fig. 4b, c). Therefore, in the follow-up investigations hereafter we focused on lung adenocarcinoma to study USP29.

Through comparing the expression levels of our panel of DUBs in lung adenocarcinoma tissues using sequencing data from the Cancer Genome Atlas (TCGA) retrieved from the cBio Cancer Genomics Portal (http://cbioportal.org), the mRNA levels of USP29 appeared to represent one belongs to the lowest abundance group (Fig. 4d). Indeed, in our endeavors to knock down USP29 expression in lung adenocarcinoma cell lines, the efficiency remained unsatisfactory with numerous attempts using siRNAs and shRNAs. We therefore switched to the opposite approach of overexpression and thus generated H1299 and H1975 adenocarcinoma cell lines stably overexpressing USP29, which displayed consistent upregulation of Snail1 levels (Fig. 4e).

**Fig. 5:**
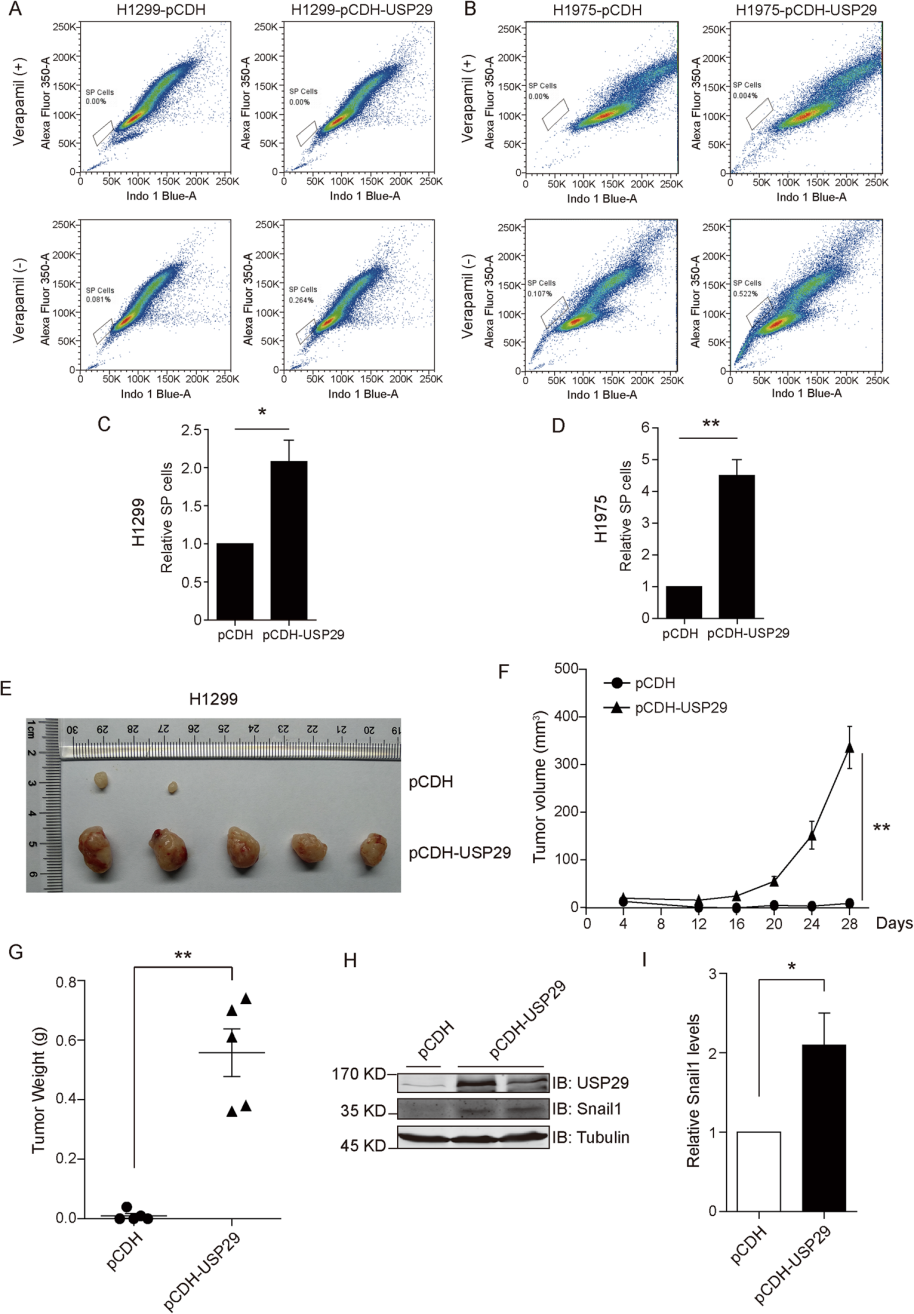
USP29 expression enhances stemness of lung adenocarcinoma cells and in vivo tumorigenesis in xenograft mouse model. **a**, **b** Side population was identified in H1299 and H1975 cells stably transfected with control or USP29-expressing vectors with the presence and absence of verapamil (200 μM). Scatter plots show representative flow cytometric analysis from three independent experiments for H1299 and H1975 as indicated, with solid boxes indicating side population cells. **c**, **d** The relative quantification of side population cells from (**a**, **b**), respectively (*n* = 3). **e** The capabilities of H1299-pCDH and H1299-pCDH-USP29 stable cell lines to form tumor xenograft in athymic nude mice were compared in parallel, with the image showing resected xenograft tumors. **f** The volumes of tumor xenograft formed by two cell lines during the experimental period were measured and plotted. **g** Weights of tumor xenografts resected were measured and plotted. **h** Tumor xenograft tissues were processed for Western blotting analysis to probe for USP29 and Snail1 levels. Tubulin blot shows equal loading. **i** Quantification data from (**h**). All error bars represent the standard error of the mean (SEM), with *p value < 0.05 and **p value < 0.01.

One predominant role that Snail1 plays in cancer cells is the conferment of mesenchymal characteristics and cancer stem cell traits. To evaluate the competence of USP29 in provoking lung adenocarcinoma cells into acquiring mesenchymal features and enhancing cell stemness, we conducted phenotypic assays to examine the spheroid formation and migration of H1299 and H1975 cells stably overexpressing USP29. Our observations revealed that the capabilities of cells to migrate and form spheroids were substantially potentiated in the presence of USP29 overexpression (Fig. 4f–i). Furthermore, in subsequent flow cytometric analyses using verapamil to distinguish cell populations associated with cancer stem cell-like characteristics, both H1299 and H1975 cells stably expressing USP29 showed significantly increased proportions in these side populations as compared to their respective control samples (Fig. 5a–d).

In light of the recognized notion that the stemness of cancer cells positively correlate with the tumorigenic potentials in vivo, we evaluated the influence of USP29 overexpression on H1299 xenograft growth in nude mice. In doing this, the same quantities of H1299 cells stably transfected with control or USP29-expressing vectors were subcutaneously inoculated into athymic nude mice to generate tumor xenografts. Recorded xenograft growth revealed dramatically accelerated increases in tumor volumes incurred by USP29 overexpression, recalling the stimulatory impact of USP29 on tumor spheroid formation (Fig. 5e–g). Consistently, Western blotting analysis of tumor xenograft samples confirmed elevated Snail1 levels in samples with enhanced USP29 expression (Fig. 5h, i). As a whole, our collective findings from both in vitro and in vivo investigations suggest that USP29 upregulation appears to be efficient in enhancing cancer stem cell-associated characteristics in lung adenocarcinoma cells.

### Cancer cells hijack the USP29-Snail1 axis to facilitate chemoresistance

**Fig. 6:**
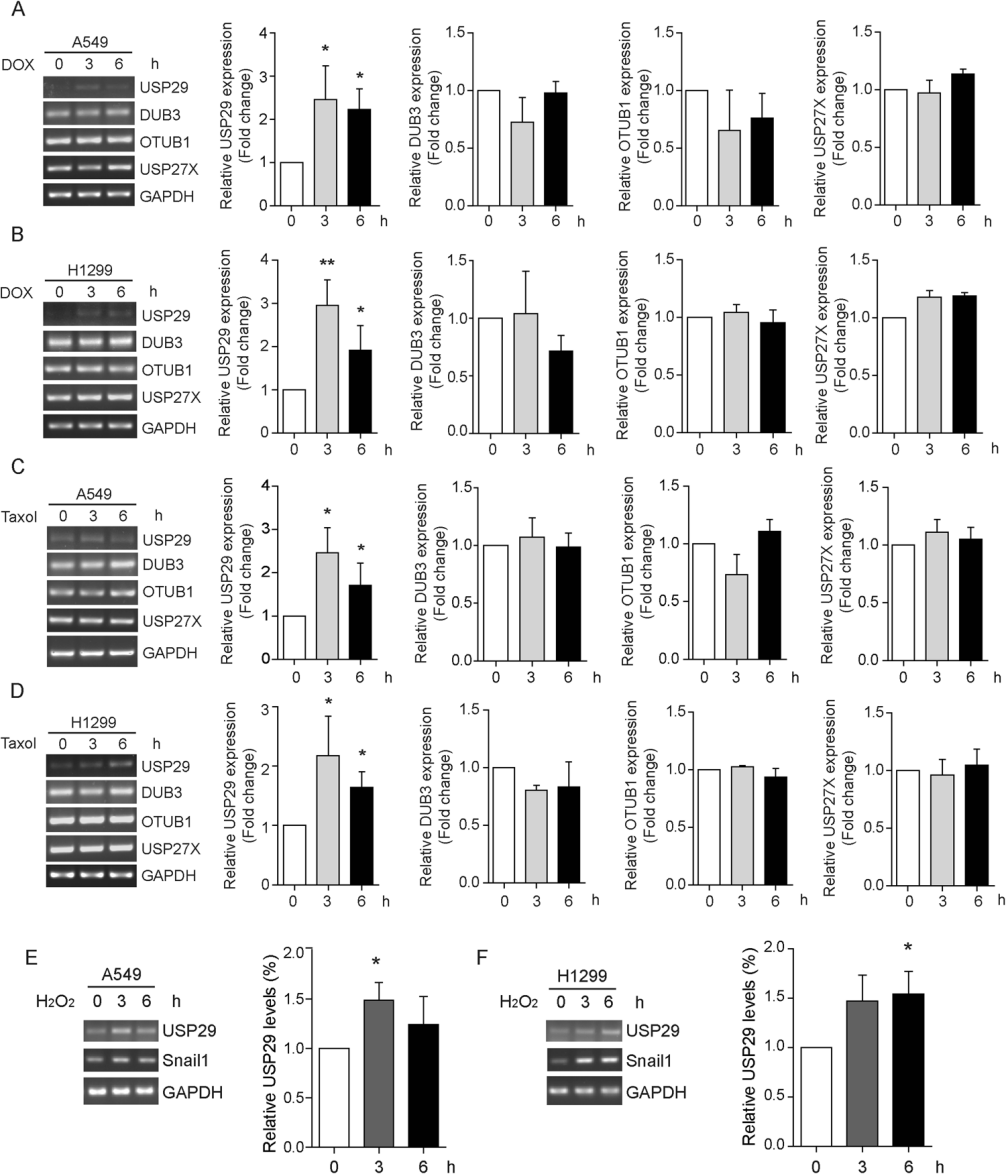
USP29 is transcriptionally activated in response to chemotherapy and oxidative stress in lung adenocarcinoma cells. **a**–**d** A549 and H1299 cells were treated with doxorubicin (DOX, 5 μg/ml) and taxol (10 μM) separately as indicated for 3 and 6 h, prior to RNA extraction and cDNA preparation by RT-PCR. Relative mRNA levels of USP29, DUB3, OTUB1, and USP27X were assessed by carrying out PCR assays with specific primer sets. GAPDH mRNA was detected as control. Images on the left show representative results from three independent experiments. Column charts on the right show quantification data of the relative mRNA amounts of the four DUBs (*n* = 3). **e**, **f** Cultured A549 and H1299 cells were treated with hydrogen peroxide at 2 mM for 1 h before recovery in normal culture media. After indicated times, cells were harvested to extract total RNAs. cDNA was prepared by reverse transcription and used in PCR assays to examine the mRNA levels of USP29 and Snail1 at various conditions. Images show representative results from three independent assays, with column charts showing the quantification data of relative USP29 mRNA abundance (*n* = 3). All error bars represent the standard error of the mean (SEM), with *p value < 0.05 and **p value < 0.01.

Considering the pivotal roles that Snail1 plays during the development of resistance to chemotherapies, we posited that the USP29-Snail1 axis might be exploited by cancer cells in their acquisition of chemoresistance. To this end, we treated A549 and H1299 lung adenocarcinoma cells with doxorubicin and paclitaxel, two chemotherapeutic agents routinely used in the clinical treatment of lung cancer, and examined the changes in mRNA levels of USP29 along with those of three other DUBs (DUB3, OTUB1, and USP27X) reported to stabilize Snail1. Following chemotherapy treatments, USP29 mRNA levels were observed to be evidently upregulated in both A549 and H1299 cells, while those of DUB3, OTUB1, and USP27X mRNAs recorded no significant induction in either cell line (Fig. 6a–d). These findings indicate a unique feature of USP29 to respond to chemotherapeutic agents among various Snail1 DUBs in lung adenocarcinoma cells.

**Fig. 7:**
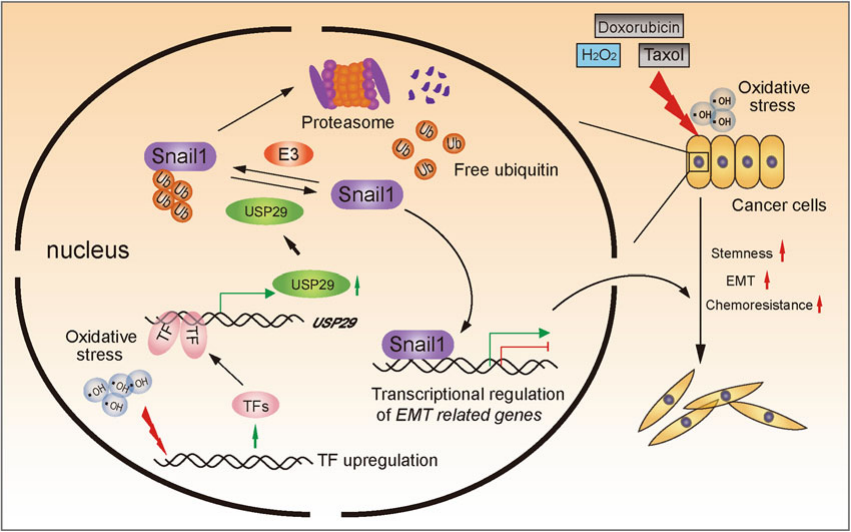
Schematic diagram depicting the oxidative stress-USP29-Snail1 regulation axis of cancer cells in responsive to chemotherapies. Our current working model proposes that lung adenocarcinoma cells display their intrinsic capabilities of rewiring transcriptional circuitry to increase USP29 expression in responsive to oxidative stresses incurred by chemotherapeutic agents. USP29 upregulation leads to the stabilization of Snail1, which acts as the master regulator to enable the expression of a range of mesenchymal genes. As a consequence, this oxidative stress-USP29-Snail1 regulation axis confers cancer cells enhanced stemness and chemoresistance. TF transcription factor, Ub ubiquitin, E3 ubiquitin E3 ligase.

It has been widely recognized that chemotherapeutic agents incur oxidative stresses in cancer cells^36^. Furthermore, hydrogen peroxide-induced oxidative stresses have been shown to regulate USP29 expression^37^. Therefore, we next sought to investigate the influence of cellular oxidative stresses on USP29 expression in lung adenocarcinoma A549 and H1299 cells. As shown in Fig. 6e, f, USP29 mRNA levels effectively responded to hydrogen peroxide treatment, which was in accordance with observations from Levens and colleagues, thus providing a mechanistic explanation for the chemotherapy-induced upregulation of USP29 in non-small cell lung cancer^37^. Taken together, our findings unravel an oxidative stress-induced USP29-Snail1 regulatory pathway integrated by lung adenocarcinoma cells in response to chemotherapies to acquire cancer stem cell-like characteristics and develop drug resistance (Fig. 7).

## Discussion

Lung cancer has remained a leading cause of cancer-associated deaths and recorded steady increases in annual mortality globally over decades^38,39^. Statistical analysis estimates over 220,000 new cases of diagnoses (lung and bronchus) and deaths of patients with lung and bronchus cancers exceeding 140,000 in 2019 alone in the United States^40^. Hence, lung cancer has posed formidable challenges to human health, and correspondingly enormous efforts have been devoted to developing effective therapeutic regimens. Although a proportion of patients would benefit from recent major breakthroughs in targeted therapies and immune checkpoint-based approaches, the remainder that nevertheless represents a greater portion will likely go through routine chemotherapy-based treatments^41^. In spite of the sensitivity that tumor cells initially show in response to chemotherapy agents, cancer ultimately relapses with acquired drug resistance in most cases and finally becomes incurable. Hence, it is of utmost importance to preclude the occurrence of chemoresistance in the clinical management of lung cancer.

From a subcellular localization-directed screening of DUBs, we identified USP29 as a novel regulator of Snail1 levels. Despite relatively low expression levels, USP29 exhibited the most potent stabilizing effect towards Snail1 expression. This DUB post-translationally elevated Snail1 levels through counteracting its ubiquitylation-mediated proteasomal degradation, in which process the DUB activity of USP29 was a necessity. Bioinformatic analysis revealed a reverse correlation between USP29 expression and patient prognoses in lung adenocarcinoma. Such tumor-promoting effects of USP29 were experimentally validated through a series of in vitro and in vivo phenotypic assays, with several lines of evidence in support of the notion that USP29 expression positively correlated with the enhanced stemness of lung adenocarcinoma cells.

It is intriguing that certain labile transcription factors can be stabilized by diverse DUBs, with p53 showing a leading example. Since the identification of herpes virus-associated ubiquitin-specific protease (HAUSP, officially known as USP7) as the first DUB that regulates p53 stability over a decade ago, the growing list of p53 DUBs has hitherto expanded to comprise more than 10 DUBs, including members from USP, OTU, and Josephin families^42,43^. Although the pathological implications and specific functionality of the p53-stabilizing effects of these DUBs require further elaboration, these findings start to disclose the potential spatial and/or temporal requirements of p53 for the involvements of various DUB activities. Likewise, several DUBs including DUB3, OTUB1, USP1, and USP27X have been reported to be capable of stabilizing Snail1 and promoting cancer progression, which also indicates the preference of Snail1 towards different DUBs within assorted cellular contexts and milieus^4,19–22^. Specifically, DUB3 was shown to respond to IL-6-stimulated transcriptional activation and stabilize Snail1 in breast cancer cells; while USP27X expression was reported to be induced by TGFβ, which assisted the upregulation of Snail1 and other mesenchymal genes^20,22^.

In the present study, we disclose USP29 as a potent Snail1 stabilizer that is distinct from other Snail1 DUBs by responding to oxidative stresses, which are frequently incurred by chemotherapies. *USP29* has been revealed as a paternally expressed gene that locates in the imprinted *PEG3* (Paternally expressed gene 3) domain^44^. The upstream sequence of *USP29* contains binding sites for the transcription factor Yin Yang 1 (YY1) in the paternal chromosome, but in the maternal copy methylation of CpG site in this region abrogates YY1-mediated transcription^45,46^. Interestingly, several lines of evidence have disclosed enhanced YY1 activity in response to oxidative stress conditions^47,48^. USP29 likely represents an advantageous choice of cancer cells owing to its high efficiency to stabilize Snail1 even with relatively low abundance, considering the critical challenges posed at them within chemotherapeutic milieus. It is noteworthy that oxidative stress conditions also directly elevate the transcription of Snail1, therefore the upregulation of Snail1 levels during oxidative stresses seems to be coordinated at multiple facets. Till now, only limited substrates of USP29 have been unveiled, which include p53 and the checkpoint adaptor Claspin^37,49^. Considering the evidence that USP29 is expressed at low levels under steady-state conditions, the requirements of USP29 DUB activity by its extra potential substrates might be revealed under specific circumstances such as oxidative stress conditions.

Being a cysteine protease, USP29 might serve as a potential therapeutic target for pharmacological intervention^50^. It is tempting to speculate that the inhibition of USP29 DUB activity will interfere with Snail1 levels and subsequently deter the development of chemoresistance and metastasis in a variety of cancers. Furthermore, considering the evidence that the *USP29* transcript in cow has lost protein-coding capability, together with recent investigation on *USP29* knockout mice that revealed no evident abnormalities, the utilization of USP29 small molecule inhibitors might benefit from limited side effects^51,52^. Hence, the current working model of the oxidative stress-USP29-Snail1 regulation axis holds potential for future translational applications and warrants further investigations.

## Supplementary information

Supplementary Table 1

## References

[1] Shaked Y (2016). Balancing efficacy of and host immune responses to cancer therapy: the yin and yang effects. Nat. Rev. Clin. Oncol..

[2] Keklikoglou I (2019). Chemotherapy elicits pro-metastatic extracellular vesicles in breast cancer models. Nat. Cell Biol..

[3] Chang YS, Jalgaonkar SP, Middleton JD, Hai T (2017). Stress-inducible gene Atf3 in the noncancer host cells contributes to chemotherapy-exacerbated breast cancer metastasis. Proc. Natl Acad. Sci. USA.

[4] Sonego M (2019). USP1 links platinum resistance to cancer cell dissemination by regulating Snail stability. Sci. Adv..

[5] Karagiannis, G. S. et al. Neoadjuvant chemotherapy induces breast cancer metastasis through a TMEM-mediated mechanism. *Sci. Transl. Med.*10.1126/scitranslmed.aan0026 (2017). 10.1126/scitranslmed.aan0026 PMC5592784 28679654

[6] Moreno-Bueno G, Portillo F, Cano A (2008). Transcriptional regulation of cell polarity in EMT and cancer. Oncogene.

[7] Baulida J, Garcia de Herreros A (2015). Snail1-driven plasticity of epithelial and mesenchymal cells sustains cancer malignancy. Biochimica et. Biophysica Acta.

[8] Yang J, Weinberg RA (2008). Epithelial-mesenchymal transition: at the crossroads of development and tumor metastasis. Dev. Cell.

[9] Thiery JP, Acloque H, Huang RY, Nieto MA (2009). Epithelial-mesenchymal transitions in development and disease. Cell.

[10] Turley EA, Veiseh M, Radisky DC, Bissell MJ (2008). Mechanisms of disease: epithelial-mesenchymal transition–does cellular plasticity fuel neoplastic progression?. Nat. Clin. Pract. Oncol..

[11] Vega S (2004). Snail blocks the cell cycle and confers resistance to cell death. Genes Dev..

[12] Nieto MA, Huang RY, Jackson RA, Thiery JP (2016). Emt: 2016. Cell.

[13] Mani SA (2008). The epithelial-mesenchymal transition generates cells with properties of stem cells. Cell.

[14] Diaz VM, de Herreros AG (2016). F-box proteins: Keeping the epithelial-to-mesenchymal transition (EMT) in check. Semin. Cancer Biol..

[15] Diaz VM, Vinas-Castells R, Garcia de Herreros A (2014). Regulation of the protein stability of EMT transcription factors. Cell Adhes. Migr..

[16] Vinas-Castells R (2010). The hypoxia-controlled FBXL14 ubiquitin ligase targets SNAIL1 for proteasome degradation. J. Biol. Chem..

[17] Zheng H (2014). PKD1 phosphorylation-dependent degradation of SNAIL by SCF-FBXO11 regulates epithelial-mesenchymal transition and metastasis. Cancer Cell.

[18] Zhou BP (2004). Dual regulation of Snail by GSK-3beta-mediated phosphorylation in control of epithelial-mesenchymal transition. Nat. Cell Biol..

[19] Liu T (2017). CDK4/6-dependent activation of DUB3 regulates cancer metastasis through SNAIL1. Nat. Commun..

[20] Wu Y (2017). Dub3 inhibition suppresses breast cancer invasion and metastasis by promoting Snail1 degradation. Nat. Commun..

[21] Zhou H (2018). OTUB1 promotes esophageal squamous cell carcinoma metastasis through modulating Snail stability. Oncogene.

[22] Lambies, G. et al. TGFbeta-activated USP27X deubiquitinase regulates cell migration and chemoresistance via stabilization of Snail1. *Cancer Res.*10.1158/0008-5472.CAN-18-0753 (2018). 10.1158/0008-5472.CAN-18-0753 PMC9386731 30341066

[23] Komander D, Clague MJ, Urbe S (2009). Breaking the chains: structure and function of the deubiquitinases. Nat. Rev. Mol. Cell Biol..

[24] Clague MJ, Coulson JM, Urbe S (2012). Cellular functions of the DUBs. J. Cell Sci..

[25] Clague MJ, Urbé S, Komander D (2019). Breaking the chains: deubiquitylating enzyme specificity begets function. Nat. Rev. Mol. Cell Biol..

[26] Urbe S (2012). Systematic survey of deubiquitinase localization identifies USP21 as a regulator of centrosome- and microtubule-associated functions. Mol. Biol. Cell.

[27] Clague MJ (2013). Deubiquitylases from genes to organism. Physiological Rev..

[28] Vinas-Castells R (2014). Nuclear ubiquitination by FBXL5 modulates Snail1 DNA binding and stability. Nucleic Acids Res..

[29] Li L, Zhou H, Zhu R, Liu Z (2019). USP26 promotes esophageal squamous cell carcinoma metastasis through stabilizing Snail. Cancer Lett..

[30] Sowa ME, Bennett EJ, Gygi SP, Harper JW (2009). Defining the human deubiquitinating enzyme interaction landscape. Cell.

[31] Wang T (2018). The exon 19-deleted EGFR undergoes ubiquitylation-mediated endocytic degradation via dynamin activity-dependent and -independent mechanisms. Cell Commun. Signal.

[32] Zhang Y (2016). Neratinib induces ErbB2 ubiquitylation and endocytic degradation via HSP90 dissociation in breast cancer cells. Cancer Lett..

[33] Cerami E (2012). The cBio cancer genomics portal: an open platform for exploring multidimensional cancer genomics data. Cancer Discov..

[34] Gao J (2013). Integrative analysis of complex cancer genomics and clinical profiles using the cBioPortal. Sci. Signal..

[35] Gyorffy B, Surowiak P, Budczies J, Lanczky A (2013). Online survival analysis software to assess the prognostic value of biomarkers using transcriptomic data in non-small-cell lung cancer. PloS ONE.

[36] Tiligada E (2006). Chemotherapy: induction of stress responses. Endocr.-Relat. Cancer.

[37] Liu J (2011). JTV1 co-activates FBP to induce USP29 transcription and stabilize p53 in response to oxidative stress. EMBO J..

[38] Mortality GBD, Causes of Death C (2015). Global, regional, and national age-sex specific all-cause and cause-specific mortality for 240 causes of death, 1990–2013: a systematic analysis for the Global Burden of Disease Study 2013. Lancet.

[39] Bray F (2018). Global cancer statistics 2018: GLOBOCAN estimates of incidence and mortality worldwide for 36 cancers in 185 countries. CA.

[40] Siegel RL, Miller KD, Jemal A (2019). Cancer statistics, 2019. CA.

[41] Camidge, D. R., Doebele, R. C. & Kerr, K. M. Comparing and contrasting predictive biomarkers for immunotherapy and targeted therapy of NSCLC. *Nat. Rev. Clin. Oncol.*10.1038/s41571-019-0173-9 (2019). 10.1038/s41571-019-0173-9 30718843

[42] Li M (2002). Deubiquitination of p53 by HAUSP is an important pathway for p53 stabilization. Nature.

[43] Kwon SK, Saindane M, Baek KH (2017). p53 stability is regulated by diverse deubiquitinating enzymes. Biochimica et. biophysica acta.

[44] Kim J (2000). Discovery of a novel, paternally expressed ubiquitin-specific processing protease gene through comparative analysis of an imprinted region of mouse chromosome 7 and human chromosome 19q13.4. Genome Res..

[45] Kim J, Kollhoff A, Bergmann A, Stubbs L (2003). Methylation-sensitive binding of transcription factor YY1 to an insulator sequence within the paternally expressed imprinted gene, Peg3. Hum. Mol. Genet..

[46] He H, Ye A, Perera BPU, Kim J (2017). YY1’s role in the Peg3 imprinted domain. Sci. Rep..

[47] Morozzi G (2017). Oxidative stress-induced S100B accumulation converts myoblasts into brown adipocytes via an NF-kappaB/YY1/miR-133 axis and NF-kappaB/YY1/BMP-7 axis. Cell Death Differ..

[48] Liu W, Guo Q, Zhao H (2018). Oxidative stress-elicited YY1 potentiates antioxidative response via enhancement of NRF2-driven transcriptional activity: A potential neuronal defensive mechanism against ischemia/reperfusion cerebral injury. Biomed. Pharmacother. = Biomedecine pharmacotherapie.

[49] Martin Y (2015). USP29 controls the stability of checkpoint adaptor Claspin by deubiquitination. Oncogene.

[50] Pal A, Young MA, Donato NJ (2014). Emerging potential of therapeutic targeting of ubiquitin-specific proteases in the treatment of cancer. Cancer Res..

[51] Huang, Z. et al. The deubiquitinating gene Usp29 is dispensable for fertility in male mice. *Sci. China. Life Sci.*10.1007/s11427-018-9469-4 (2019). 10.1007/s11427-018-9469-4 30919279

[52] Kim J, Bergmann A, Choo JH, Stubbs L (2007). Genomic organization and imprinting of the Peg3 domain in bovine. Genomics.

