# Supplementary material for: USP29 enhances chemotherapy-induced stemness in non-small cell lung cancer via stabilizing Snail1 in response to oxidative stress
Source: Cell Death Dis. 2020 Sep 23;11(9):796. doi: 10.1038/s41419-020-03008-5 (PMC7511960; doi:10.1038/s41419-020-03008-5)
Supplement: Supplementary file 1 — Supplementary Table 1 [file 41419_2020_3008_MOESM1_ESM.docx]

Supplementary Table 1. List of primers used in this study. All sequences are given in the order of 5’ to 3’.

| *USP29* | Forward primer | AGCAGGCCTGGTTCACATAC |
| --- | --- | --- |
|  | Reverse primer | TGTGCTAGGTAGCCGAGAGT |
| *Snail1* | Forward primer | GGCCCTGGCTGCTACAAGGC |
|  | Reverse primer | CTCGAGGGTCAGCGGGGACA |
| *DUB3* | Forward primer | ACACTTTTGACCCTTACCTGG |
|  | Reverse primer | TGGAGACAAAGACCGCAATG |
| *OTUB1* | Forward primer | TGTTTCTATCGGGCTTTCGG |
|  | Reverse primer | AGGTGCTCTGGTCATTGAAG |
| *USP27X* | Forward primer | AGAATCACCTCCAGCTTTACG |
|  | Reverse primer | TGCCTGTACCCTGCTAAATG |
| *GAPDH* | Forward primer | CGGATTTGGTCGTATTGGG |
|  | Reverse primer | CTGGAAGATGGTGATGGGATT |
